# All‐Inside Anterior Cruciate Ligament Reconstruction With Accurate Measurement Technique

**DOI:** 10.1002/atn2.70284

**Published:** 2026-10-19

**Authors:** Daoliang Xu, Wei Yu, Jian Lin, Renjie Li, Zhikai Lu, Yulong Zhou, Xiaoyun Pan

**Affiliations:** ^1^ Department of Orthopaedics The Second Affiliated Hospital and Yuying Children's Hospital of Wenzhou Medical University Wenzhou Zhejiang China; ^2^ Key Laboratory of Orthopedics of Zhejiang Province Wenzhou Zhejiang China; ^3^ The Second Clinical School of Medicine Wenzhou Medical University Wenzhou Zhejiang China; ^4^ Department of Orthopaedics The Third People's Hospital of Cangnan Wenzhou Zhejiang China; ^5^ Department of Orthopedics No. 906 Hospital of Joint Logistic Support Force of PLA Ningbo Zhejiang China

## Abstract

The all‐inside anterior cruciate ligament reconstruction technique is characterized by closed bone sockets for anterior cruciate ligament graft passage and typically adopts suspensory fixation instead of screw fixation. Inaccurate determination of the intra‐articular length (IAL) of the graft is associated with an elevated risk of graft “bottoming out” and excessive length consumption of the tibial sockets. Notably, the IAL is dependent on the patient's knee dimensions and intraoperative tunnel positioning, thus requiring individualized measurement. Herein, we propose an intraoperative method for accurate IAL measurement in all‐inside anterior cruciate ligament reconstruction, which enables the creation of bone sockets with appropriate lengths based on individual IAL values. This technique effectively mitigates graft‐tunnel mismatch and improves the stability of graft fixation, thereby optimizing the clinical outcomes of the all‐inside anterior cruciate ligament reconstruction.

## Graphical abstract

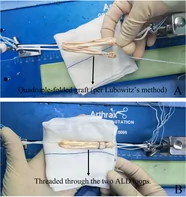

**VIDEO 1:** This surgical technique uses a simple self‐fabricated measuring device to accurately acquire key intraoperative parameters for all‐inside anterior cruciate ligament reconstruction. It provides a decisive basis for intraoperative graft preparation and the creation of tibial and femoral sockets. After induction of anesthesia, the patient is placed in the supine position, with the operative right knee flexed to 90°. The steps are as follows: 1. Insert the arthroscope via the anteromedial/anterolateral portal to assess anterior cruciate ligament injury and address concomitant lesions (e.g., meniscal tears). 2. Debride the anterior cruciate ligament femoral and tibial footprints to confirm anatomical insertion sites. 3. Create 4.5‐mm‐diameter tibial and femoral tunnels using standard techniques and measure their lengths under direct arthroscopic vision. 4. Introduce the custom measuring instrument through the anteromedial tibial tunnel's cortical exit. With the knee flexed at 30° in the posterior drawer position, measure the distance from the tibial cortex to the femoral footprint to determine the intra‐articular length. 5. Based on the intra‐articular length and tunnel lengths, create appropriately sized tibial and femoral sockets (≥20 mm bilaterally). 6. Insert the graft into the sockets, deploy the adjustable loop buttons for fixation, and adjust tension to restore anterior cruciate ligament stability. Video content can be viewed at https://doi.org/10.1002/atn2.70284.

The philosophy underlying all‐inside anterior cruciate ligament reconstruction (ACLR) centers on maximizing graft length use, coupled with closed‐socket tunnels and dual suspensory fixation. The concept was first described by Morgan et al. in 1995,^1^ and its clinical popularization was driven by Lubowitz et al.,^2^ who sequentially advanced the methodology: retrograde drilling in 2006 and a second‐generation technique integrating outside‐in femoral drilling with adjustable cortical suspensory fixation in 2011.^3^

Despite its wide application, graft‐tunnel mismatch remains a major cause of surgical failure,^4^ primarily attributed to inaccurate estimation of the intra‐articular length (IAL)—a key parameter that determines socket depth and graft tension. In principle, to prevent graft “bottoming out” during tensioning and fixation, the graft length should be shorter than the total length derived from the sum of the femoral socket length, tibial socket length, and IAL. Only by adhering to this principle can satisfactory clinical outcomes be guaranteed.^2^ ^,^ ^3^

However, a clearly defined optimal surgical protocol for contemporary all‐inside ACLR remains elusive. Critical determinants include socket depth and IAL—both of which are subject to variation based on intraoperative knee positioning and patient‐specific knee dimensions. As such, these parameters cannot be directly referenced from the anatomical values of the native anterior cruciate ligament (ACL); instead, they are individualized indices that require precise intraoperative measurement. Here, we put forward a standardized measurement technique using our custom‐designed instrument, specifically tailored to address this key clinical challenge in all‐inside ACLR.

## SURGICAL TECHNIQUE

### Arthroscopic Examination and Graft Preparation

The patient is secured in a leg holder allowing 0° to 120° knee flexion. After anesthesia induction, a full arthroscopic assessment of ACL is performed, with concomitant injuries managed as clinically indicated. Postevaluation, the arthroscope is removed, and an oblique anteromedial incision is made at the pes anserinus level. For the all‐inside ACLR, autologous semitendinosus and gracilis tendon grafts are preferred, requiring a length ≥240 mm (to be tripled or quadrupled to 65‐75 mm) and diameter ≥8 mm. Graft preparation follows Lubowitz's method^3^: both graft ends are looped over an adjustable loop device (Smith & Nephew, Andover, MA), which is a cortical suspensory fixation device commonly used in all‐inside ACLR (Figure 1). Markings are then placed on either end of the tendon corresponding to the predetermined socket depth derived from IAL measurements.

**FIGURE 1:**
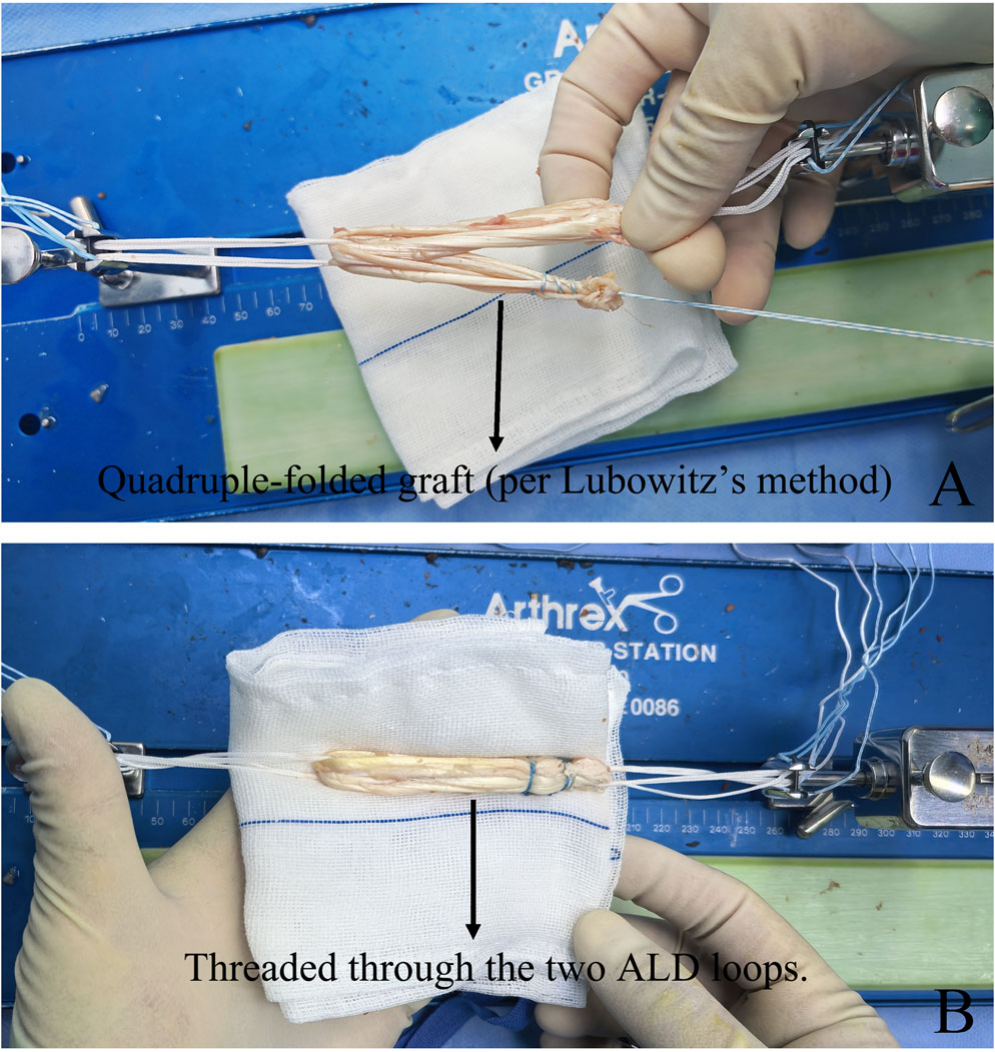
(A) Autologous semitendinosus and gracilis tendon grafts were prepared on a dedicated graft preparation platform (Graft PREP STATION, Arthrex, Naples, FL). (B) The graft was quadruple folded following Lubowitz's^3^ technique to obtain a target length of approximately 65 mm. (ALD, adjustable loop device.)

### Accurate Measurement for ACLR Parameters

After debridement of the ACL femoral footprint, the anatomic insertion site is confirmed to ensure accurate positioning. The tibial footprint is then debrided appropriately. Subsequently, the ACL guide (Arthrex, Naples, FL) is introduced and placed anatomically over the tibial footprint, with positioning verified via the anterolateral portal. A 2.0 mm guide pin is inserted, followed by drilling of a 4.5 mm tibial tunnel. Next, either a low‐profile reamer (Arthrex) or an inside‐out guide (Arthrex) is positioned at the center of the femoral footprint, and a matching 4.5 mm femoral tunnel is created in the same manner. The length of the femoral tunnel is measured using conventional techniques.

Under direct visualization and arthroscopic guidance (Figure 2), the length of the 4.5 mm tibial tunnel is measured with precision (designated as *a*). Subsequently, a traction suture is employed to guide the custom measuring device (Figure 3) through the tibial and femoral tunnels, and its zero‐point is fixed firmly to the tibial cortex (Figure 4A). With the knee held at 30° of flexion in the posterior drawer position, the total distance of the device at the femoral footprint (designated as *b*) is recorded under direct visualization (Figure 4C), and the length of the tibial tunnel is double checked (Figure 4B). The IAL (designated as *c*) is calculated as follows:

**FIGURE 2:**
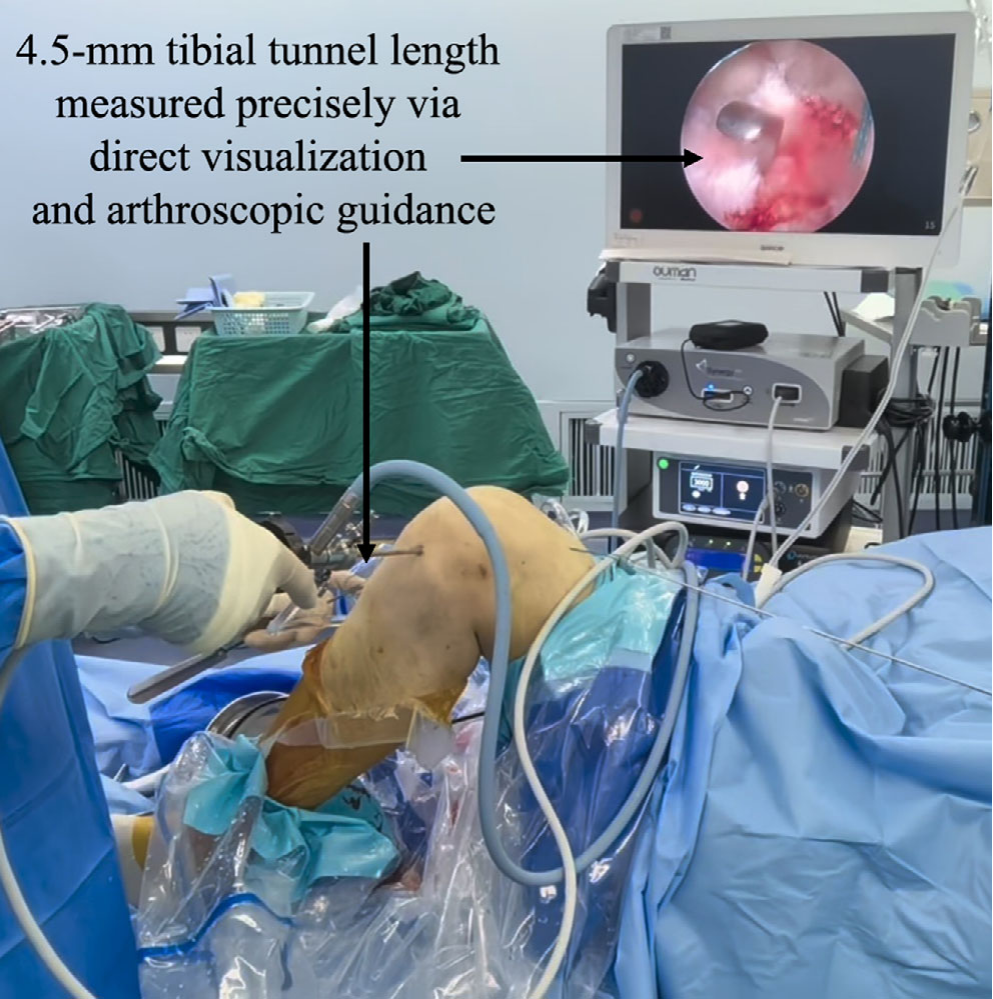
Right knee, arthroscopic view from anterolateral portal. High‐precision measurement of the tibial tunnel length was performed under combined direct visualization and arthroscopic guidance.

**FIGURE 3:**
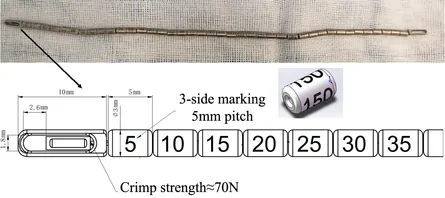
Composition of the custom measurement device for all‐inside ACLR: flexible ruler body with scale marks (ensuring measurement precision), measurement module assembled by tandem nitinol blocks (5 mm × 3 mm, length × diameter; facilitating tunnel navigation), and bilateral crimped fixation components (fixation strength ~70 N, ensuring stability during measurement). (ACLR, anterior cruciate ligament reconstruction.)

**FIGURE 4:**
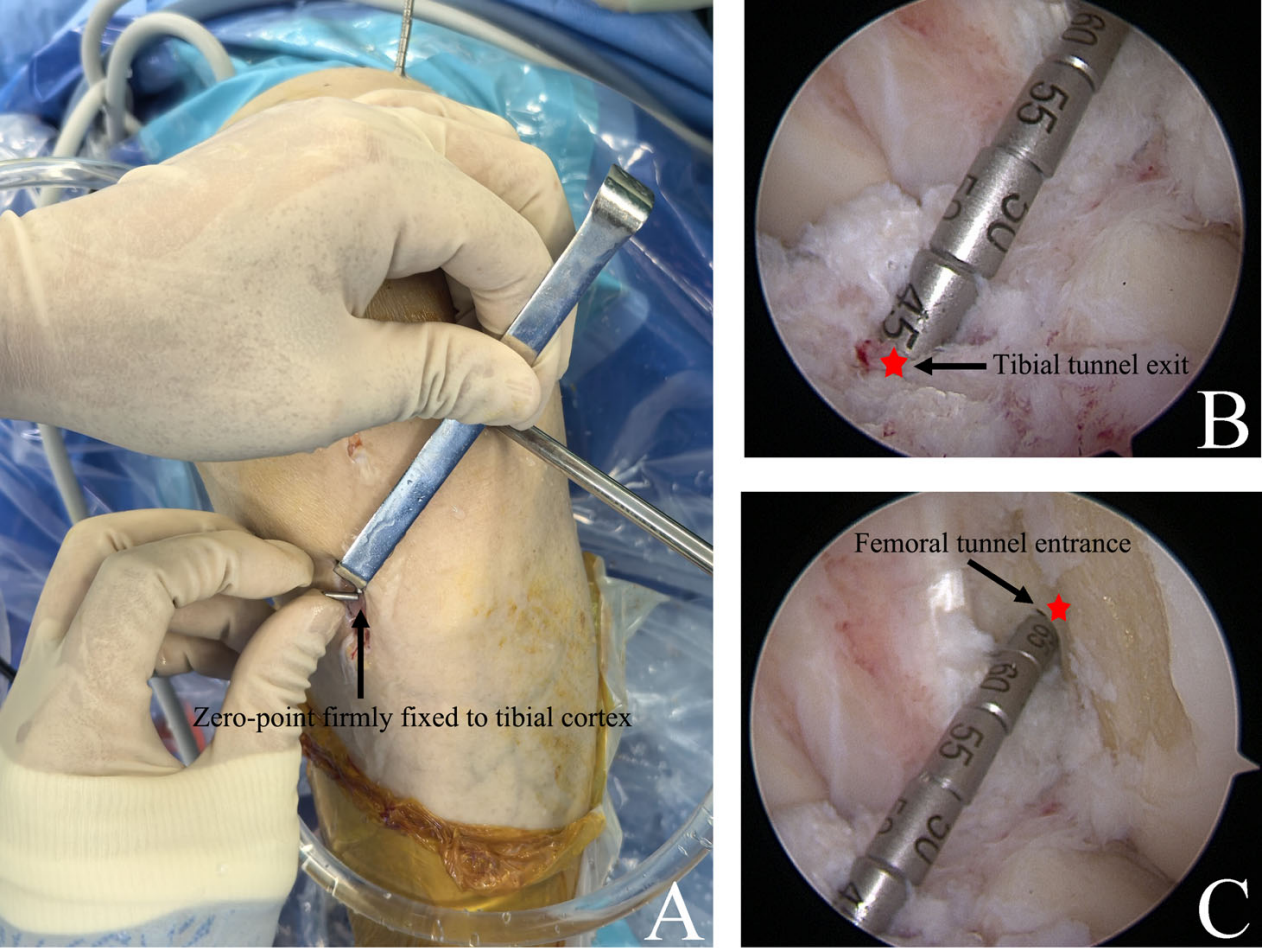
Measurement and documentation of tibial/femoral footprint distances under direct visualization. (A) Device zero‐point: firm fixation on tibial cortex (arrow). Then, the knee positioned at 30° flexion (posterior drawer position); visualized via anterolateral portal. (B,C) Asterisks indicate tibial tunnel exit and femoral tunnel entrance, aligned with 40 and 65 mm device positions.

c = b − a

### Accurate Creation of Bone Sockets

Using the measured IAL and the preferred graft length as a reference, optimal femoral and tibial socket dimensions are determined, with each socket requiring a minimum length of 20 mm. Simultaneously, the graft is marked at its tibial and femoral ends based on precalculated socket lengths. A key principle applies: the total length of the tibial and femoral socket plus IAL must exceed the overall graft length to prevent graft bottoming within sockets.^2^

The tibial socket is then drilled as planned with a second‐generation retrograde drill (FlipCutter, Arthrex, Naples, FL), using clockwise rotation and retrograde pressure. The femoral socket may be prepared via the standard technique or an outside‐in approach with the same FlipCutter, following the same method as tibial socket creation. Subsequently, passing sutures for both sockets are retrieved through the anteromedial portal and inspected to exclude soft tissue entrapment or suture entanglement.

### Achievement of Appropriate Graft Tension

First, the graft construct is inserted into the tibial socket, with arthroscopic visualization provided via the anteromedial portal. The tibial adjustable loop button is advanced until it protrudes through the anteromedial tibial cortex (Figure 5), after which the tibial end of the graft is fixed securely inside the socket (Figure 6A). Next, the femoral end of the graft is pulled into its corresponding socket until the femoral adjustable loop button is flipped over. Under direct arthroscopic view, additional tension is applied to the femoral side until the graft is seated at the premarked depth (Figure 6B). Subsequently, the knee is placed at 30° of flexion in the posterior drawer position, and the tibial adjustable loop button is deployed to attain final graft tensioning (Figure 6C). The intra‐articular graft is palpated to verify that ACL laxity has been satisfactorily alleviated (Figure 7; Video 1).

**FIGURE 5:**
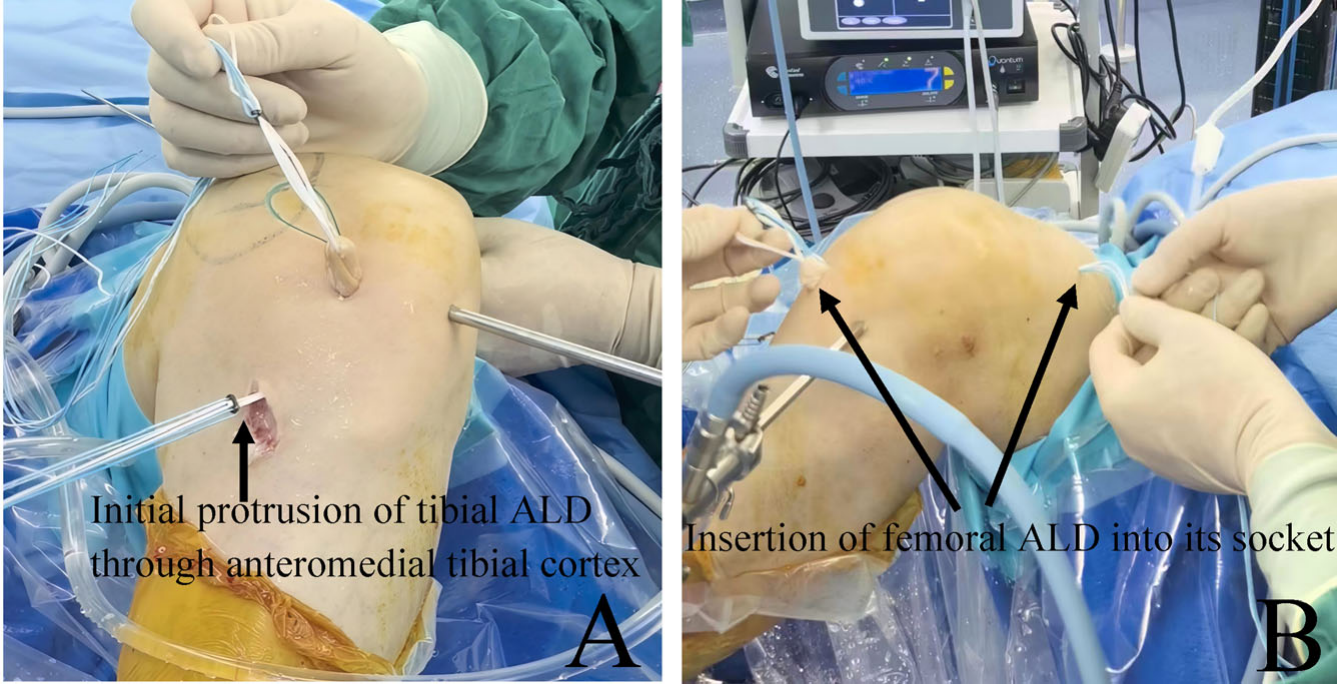
(A) Graft construct inserted into the tibial socket (anteromedial portal, arthroscopic visualization). Tibial adjustable loop button (arrow) advanced to protrude through anteromedial tibial cortex. (B) Then, the femoral end of the graft (left arrow) was pulled into its corresponding socket until the femoral adjustable loop button was flipped over. (ALD, adjustable loop device.)

**FIGURE 6:**
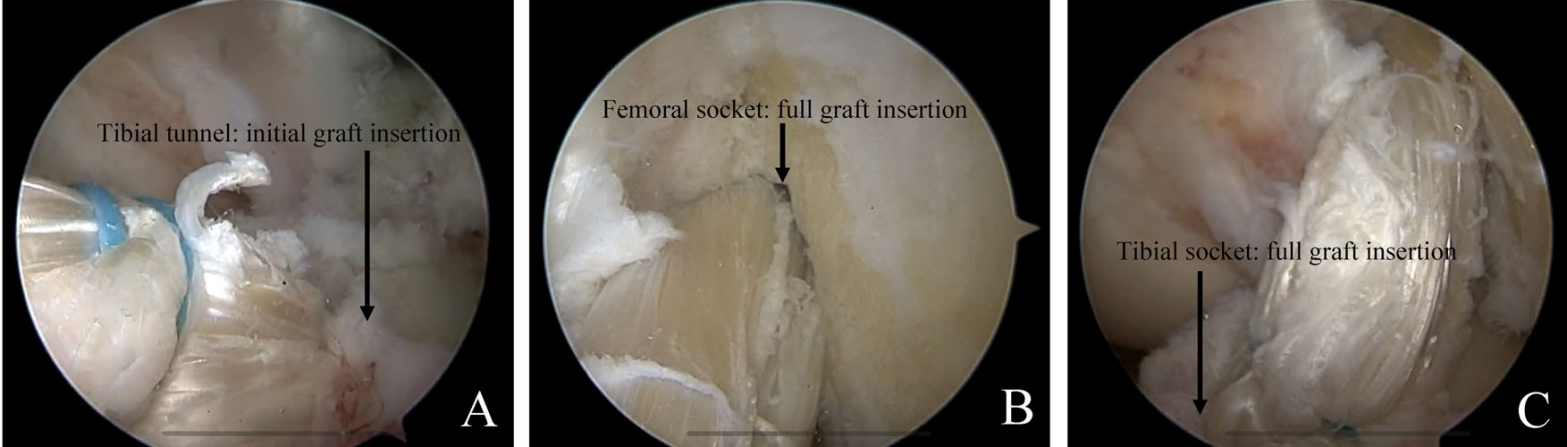
Intraoperative arthroscopic views: graft placement and tensioning. (A) Anterolateral portal view, initial graft pulling into tibial tunnel; incomplete entry was visualized (arrow). (B) Full graft tunnel insertion via femoral adjustable loop button action (arrow). (C) Posterior drawer position (30° knee flexion): final optimal graft tension achieved with tibial adjustable loop button (arrow).

**FIGURE 7:**
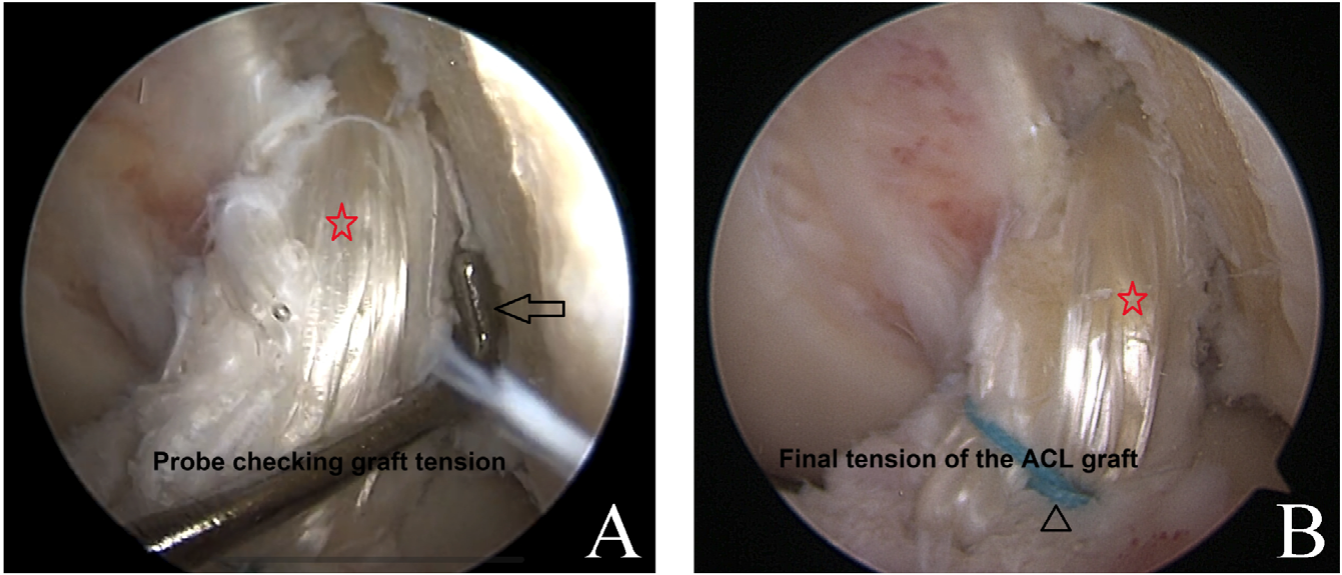
Assessment of ACL laxity. Knee flexed at 90°, viewing portal: anterolateral. (A) Arthroscopic view showing the reconstructed ACL graft (asterisk) being assessed with a probe (arrow) to verify tension and rule out residual laxity. (B) Final view showing the appropriately tensioned ACL graft (arrow). The triangular marker indicates the braided suture. (ACL, anterior cruciate ligament.)

To boost the safety and reproducibility of the abovementioned all‐inside ACLR steps—particularly for surgeons with limited experience in this approach—we have summarized core operational tips and common intraoperative risks for 3 key procedures based on our clinical practice. These factors directly impact graft fixation stability, measurement precision, and postoperative knee kinematics and are elaborated in Table 1.

**TABLE 1:** Pearls and Pitfalls

**Step**	**Pearls**	**Pitfalls**
Graft preparation	Mark graft ends with lines corresponding to precalculated socket depths; ensure folded graft length ≥65 mm and diameter ≥8 mm	Excessive marker penetration into the tibial socket may cause impingement on cancellous bone or knee subluxation
Measurement of the tibial 4.5 mm tunnel	Ensure adequate exposure of the tibial footprint; perform measurement under combined direct and arthroscopic visualization	Soft tissue obstruction at the tunnel entrance leads to overestimation of tunnel length
Measurement of the IAL	Fix the device's zero‐point firmly to the tibial cortex; maintain knee at 30° flexion in posterior drawer position during measurement	Tissue obstruction or improper knee positioning results in underestimation of IAL

IAL, intra‐articular length.

When autograft tissue lacks sufficient length or thickness for the all‐inside technique, supplementation with allograft tissue or suture tape can be a feasible alternative.

## DISCUSSION

The all‐inside ACLR is distinguished by closed femoral and tibial bone sockets and dual‐suspensory cortical fixation, which maximizes graft use and minimizes bone injury.^3^ ^,^ ^5^, ^6^, ^7^ Previous studies have reported equivalent or favorable clinical and biomechanical outcomes compared with conventional transtibial ACLR.^6^ ^,^ ^8^, ^9^, ^10^, ^11^, ^12^

However, graft tunnel mismatch and graft “bottoming out” remain leading causes of suboptimal outcomes, largely because of inaccurate intraoperative estimation of IAL. IAL varies with patient anatomy and tunnel positioning and thus cannot be simply extrapolated from native ACL measurements. Individualized, precise IAL measurement is therefore critical to determine appropriate socket depths and prevent graft overtensioning or impingement. Prior IAL measurement methods, such as marked shuttle sutures^4^ or standard depth gauges,^13^ are limited by low precision, off‐center placement, or residual tissue interference. These drawbacks lead to measurement errors and suboptimal socket preparation.

Our proposed technique addresses this flaw by enabling precise intraoperative IAL quantification, which serves as the basis for individualized socket creation. Compared with existing methods^4^ (e.g., marked shuttle sutures), our custom‐designed measuring device offers two key advantages: (1) clear scale markings and smooth navigation through 4.5 mm full‐length tunnels, ensuring centering over the femoral and tibial ACL footprints, and (2) elimination of errors caused by residual ACL stumps via the formula *c* = *b* − *a*, achieving higher measurement accuracy. Notably, precise quantification of IAL, integrated with the length of the weaved autologous graft, enables accurate modulation of tibial and femoral socket depth. This advantage effectively mitigates the “bottoming‐out” effect while preserving appropriate graft tension and further extends the applicability of the all‐inside ACLR technique to pediatric knee joints, which require shorter sockets for successful implementation of the procedure. In addition, measurement of the 4.5 mm tunnel is also essential, even in all‐inside ACLR procedures where IAL quantification is not performed. In the event of graft “bottoming out,” at least one socket requires revision to extend its length. For the purpose of ensuring stable fixation of the adjustable loop button, precise determination of the 4.5 mm tibial or femoral tunnel length is critical—a prerequisite for preventing complete cortical penetration and preserving a minimum cortical bone thickness of ≥7 mm at the tunnel interface.^14^, ^15^, ^16^

Currently, no consensus standard exists regarding the optimal socket length for ACL reconstruction. Animal experimental data have shown that at least 15 mm of graft engagement within a dense bone tunnel is essential to achieve satisfactory tendon‐bone healing.^17^ Lubowitz et al.^2^ proposed that the graft's IAL might range from roughly 29 to 36 mm, which was comparable to our measured IAL values spanning 22 to 35 mm. Drawing on these research outcomes, we advocate for a socket length of ≥20 mm bilaterally, a specification that necessitates a folded autograft length of ≥65 mm. For patients diagnosed with osteoporosis, the 4.5 mm tunnel length warrants appropriate augmentation. Additionally, to optimize graft stability, we typically construct the tibial socket 5 mm longer to reduce the risk of graft laxity.

In conclusion, precise intraoperative IAL measurement improves socket planning and graft fixation in all‐inside ACLR. This simplified, reproducible technique reduces complications and expands indications to more challenging patients. Concurrently, it facilitates the optimal use of shorter autologous tendon grafts. Limitations of this technique include the need for custom instrumentation, slightly increased operating time, and lack of long‐term clinical and biomechanical validation. The measuring device itself remains to be optimized for broader compatibility. A comprehensive overview of the technique's merits and drawbacks is provided in Table 2.

**TABLE 2:** Advantages and Disadvantages

**Advantages**	**Disadvantages**
Intuitive measuring device and easily mastered procedure	Incomplete optimization of the measurement device
Enhanced safety profile of all‐inside ACLR in pediatric/osteoporotic populations	Unvalidated long‐term biomechanical and clinical outcomes
Optimized autologous graft utility through precise IAL assessment	Prolonged operative time (requires technical proficiency to mitigate)
Mitigated graft “bottoming out” and “bungee effect”	
Reduced postoperative pain because of precise socket sizing (minimized bone trauma)	

ACLR, anterior cruciate ligament reconstruction; IAL, intra‐articular length.

## DISCLOSURES

The authors (D.X., X.P.) declare the following financial interests/personal relationships, which may be considered as potential competing interests: D.X. receives report from The Second Affiliated Hospital and Yuying Children's Hospital of Wenzhou Medical University and has patent pending. X.P. reports article publishing charges and writing assistance were provided by The Second Affiliated Hospital and Yuying Children's Hospital of Wenzhou Medical University and has patent pending. The other authors (W.Y., J.L., R.L., Z.L., Y.Z.) declare that they have no known competing financial interests or personal relationships that could have appeared to influence the work reported in this article.
